# The Role of Neighbourhood and Workplace Ethnic Contexts in the Formation of Inter-ethnic Partnerships: A Native Majority Perspective

**DOI:** 10.1007/s10680-019-09528-x

**Published:** 2019-05-27

**Authors:** Leen Rahnu, Allan Puur, Tom Kleinepier, Tiit Tammaru

**Affiliations:** 1 Centre for Migration and Urban Studies, University of Tartu, Tartu, Estonia; 2 Estonian Institute for Population Studies, Tallinn University, Tallinn, Estonia; 3 OTB - Faculty of Architecture and Built Environment, Delft University of Technology, Delft, The Netherlands

**Keywords:** Ethnically mixed partnership, Event history analysis, Segregation, Workplace, Neighbourhood, Finland, Register data

## Abstract

Although inter-ethnic encounters take place in multiple domains of daily life, ethnic intermarriage has typically been studied in relation to places of residence but rarely in relation to workplaces. Focussing on migrants is the most common approach to the study of intermarriage, whereas focussing on native majority population is less frequent. This study investigates an extent to which the share of immigrants at the workplace establishment and in the residential neighbourhood influences the natives’ likelihood of choosing a foreign-born partner. The analysis is based on longitudinal register data that cover all residents of Finland in 1999–2014. We focus on native Finnish women and men born from 1981 to 1995. We estimated a discrete-time event history model with competing risks, distinguishing the first-partnership formation with a foreign-born partner and a native-born partner. The share of immigrants in the residential neighbourhood and workplace both increase the propensity of choosing a foreign-born partner, but the share of immigrants in workplace tends to have a stronger bearing on the partner choice. High exposure to other ethnic groups in one domain is associated with reduced effect of the additional exposure occurring in another domain. The effect of ethnic diversity at workplace tends to be more pronounced among women. The study contributes to the literature by examining both the independent effect of residential and workplace contexts on the formation of ethnically mixed partnership among the native majority population, as well as the interaction between the two.

## Introduction

The large inflow of immigrants to Europe over the past decades has put to test the integration capacity of the European societies. Finland has been mainly a site of intra-European migration, but in recent decades the country has been also one of the destinations of migration flows to Europe. Willingness of members of the native majority population to intermarry is often considered as the strongest indicator of immigrant acceptance and immigrant integration in the host society (Gordon 1964; Kalmijn 1998; Qian and Lichter 2007). For migrants, intermarriage is an important pathway to the social networks in the new homeland, in learning a new language, absorbing written and unwritten rules of society and establishing a position in the labour market (Kantarevic 2005; Dribe and Lundh 2008; Meng and Meurs 2009). For native majority, the formation of mixed ethnic unions hinges on factors that relate to personal characteristics and structural opportunities to meet members of the other ethnic groups. In order to understand patterns of partnering behaviour as related to opportunity structures, the metaphor of the market is often used (Blau 1977; Kalmijn 1998). The important characteristics of the marriage market relate to the size, composition and geography of ethnic groups. This paper focuses on the role of the local-level ethnic contexts in the formation of mixed ethnic unions.

Although opportunities for first-hand inter-ethnic encounters could emerge in multiple domains of daily life, intermarriages have typically been studied in relations to one domain, most often places of residence. For people who work, however, workplaces serve as crucial places of daily interaction and encounter (Lichter et al. 1991; Piper 1997; Bratter and Zuberi 2001; Rosenfeld 2002; Adams and Ghose 2003; Niedomysl et al. 2010). Given that the workplace accounts for a sizable portion of the places where partners might meet (Kalmijn and Flap 2001; Houston et al. 2005), it is important to better understand also this aspect of the opportunity structure. We aim to expand previous studies on opportunity structures by including both workplace and residential neighbourhood ethnic contexts as a potential determinant of ethnic intermarriage. Our goal is to find out whether working together with migrants at workplaces and living together with migrants at places of residence affect natives’ willingness to intermarry with migrants. Studying natives enables us to observe whether and how migration-related shifts in the local marriage market are reflected in the pattern of partner selection. This contributes to a better understanding of the factors that shape the host society’s readiness and willingness to integrate immigrants.

While much of the empirical work on intermarriage has used cross-sectional data (e.g. Hwang et al. 1997; Kalmijn and van Tubergen 2006, 2010), we contribute to the literature by using longitudinal data from the Finnish population register. The Finnish population register allows us to construct the residential neighbourhood and workplace ethnic context variables and trace the formation of inter-ethnic partnerships during the period of 1990–2014. We will first study separately the effects of living together with migrants in residential neighbourhoods and working together with migrants in workplaces, followed by a joint modelling of the effects of residential and workplace contexts on intermarriage.

## Theoretical Background

The likelihood of formation of the ethnic intermarriages depends on the opportunities to meet members of other ethnic groups on a day-to-day basis (Kalmijn and Flap 2001). The focus of the current study is in the role of the ethnic context of places of residence and work in the formation of mixed ethnic unions among natives. Research on neighbourhood effects has shown that otherwise similar individuals may experience different life careers depending on the local contexts to what they are exposed (Durlauf 2004; Andersson and Subramanian 2006; Van Ham et al. 2012). Three overlapping mechanisms could link the ethnic make-up of the residential neighbourhoods and workplace with the propensity of intermarriage: proximity effects, network effects and socialization effects (cf. Houston 2005; Strömgren et al. 2014).

The proximity effect suggests that meeting a partner closer to home is more likely because people undertake much of their daily activities, also leisure time activities, close to home (Kamenik et al. 2016). For young people and for ethnic minorities, residential neighbourhood is an especially important site of daily activities (van Kempen and Wissink 2014). For working-age people who are active at labour market, an important share of actual social interaction takes place at workplace (e.g. Baron and Bielby 1980; Tomaskovic-Devey et al. 2006). For natives, the probability to meet immigrants differs in residential neighbourhoods and workplaces since levels of ethnic residential segregation tend to be higher than levels of ethnic workplace segregation (Ellis et al. 2004; Strömgren et al. 2014). In comparing the levels of segregation of native-born and immigrant groups in the Los Angeles, Ellis et al. (2004) further demonstrated that segregation both across residential neighbourhoods and workplaces had a cumulative effect as well: Almost half of segregation in the workplace neighbourhoods was due to segregation in the residential neighbourhoods. In order to understand opportunity structures in the formation of mixed ethnic unions, there is a need to take into account both the independent role of residential and workplace contexts, as well as the interaction effect between the two.

The network and socialization effects relate to whom people interact (networks), and how they are influenced by such interactions (socialization). The social networks that people have formed both in the current as well as in the past residential and workplace contexts determine to whom they have been exposed to. For example, Rosenbaum et al. (2003) found that low-income families who were placed to better neighbourhoods within the Moving to Opportunity experiment maintained strong social ties with their former neighbours after moving. The social networks are often gendered: Women tend to interact more often with women and men with men (Hanson and Pratt 1992), whereas the social networks of women tend to be more residential neighbourhood-based unlike social networks of men that tend to be workplace-based (Moore 1990; Wang 2010). It does not necessarily imply that people marry with a neighbour or co-worker, rather it is the expose to different ethnic contexts at places of residence and work that might shape the individual preferences and lay ground for disperse interaction networks leading to potential partners.

There are competing views on how the share of immigrant population might affect native attitudes towards immigrants at places of residence and work. On the one hand, living or working together with immigrants and getting a first-hand personal experience with ethnically diverse setting might reduce prejudices and increases tolerance towards immigrants (Pettigrew and Tropp 2006). Previous studies have found that co-ethnic unions are more common among immigrants who live in ethnic neighbourhoods, and inter-ethnic unions are more likely to emerge in mixed ethnic neighbourhoods (Hwang et al. 1997; Van Tubergen and Maas 2007). However, high share of immigrants in the immediate surrounding might not lead to higher prevalence of intermarriages for two reasons. First, it might provide a basis of feeling of threat for the members of the native majority population (Blalock 1967; Quillian 1995). Second, the increase in immigrants implies that their own-group marriage market increases and it reduces the willingness to form unions with members of the native majority population. For example, in the European context, it has been found in Estonia that a large share of ethnic minorities with a long history of residence in the host country does not come along with increase in inter-ethnic marriages (Van Ham and Tammaru 2011; Puur et al. 2018).

However, the importance of preferences and social norms should not be disregarded, even when focussing on the role of structural opportunities. The latter factors are particularly relevant for inter-ethnic unions between natives and immigrants of different origins. In cases in which the cultural background of the immigrants is similar to that of the natives, partners from the latter groups might be considered more attractive, whereas immigrants with more distant origins are less likely to form partnerships with natives. Empirical support for this assertion is provided by a number of studies (Kalmijn and van Tubergen 2010; Dribe and Lundh 2011; Hannemann et al. 2018). Another useful approach to explain inter-ethnic partnerships is the status exchange theory introduced by Merton (1941) and Davis (1941). With regard to inter-ethnic partnerships, exchange theory asserts that native partners must be compensated for their position as members of the majority group. To ensure equivalence, immigrants who partner with natives are expected to have superior characteristics, such as better education, relative to their counterparts who partner within their own group. On the other hand, majority partners who accept immigrant mates are perceived to have characteristics inferior to their peers who partner endogamously. These assertions have gained support from studies of interracial marriage in the USA (Kalmijn 1993; Bankston and Henry 1999; Qian and Lichter 2001) and Brazil (Gullickson and Torche 2014).

To conclude, the opportunity structures that shape the formation of inter-ethnic unions between members of the native majority population and immigrants might relate in important ways to the residential and workplace ethnic contexts to which natives are exposed to. This can happen because of the proximity, network and socialization effects. We expect that living and working in contexts with a higher proportion of immigrants would increase the willingness to intermarry (Hypothesis 1). However, in contexts with the highest proportion of immigrants, the willingness to intermarry with migrants will be reduced (Hypothesis 2). We further assume that workplace context is more important than residential context in shaping natives’ willingness to intermarry with migrants because of the more intense daily interaction with co-workers compared to neighbours (Hypothesis 3). We will conduct a separate analysis for the partnering behaviour of Finnish men and women. We expect that residential context is more important for native women (Hypothesis 4) and workplace context is more important for native men (Hypothesis 5) for the formation of mixed ethnic unions with immigrants. Alternatively, it is possible that the expected impact of ethnic concentration disappears after controlling for contextual factors (birth cohort, size of the workplace establishment and residential neighbourhood) as well as individual characteristics (previous international experience and level of education) (Hypothesis 6). Finally, we are interested in whether the associations between residential and workplace context and inter-ethnic partnering vary across migrant groups. In light of the preference for partners with a similar background discussed earlier in this section, we assume that these associations would be more pronounced for immigrant groups originating from settings that are culturally closer to the host society (Hypothesis 7).

## The Finnish Context

Compared to other Nordic countries Finland’s experience as a receiver of international migration is more recent. However, Finland has a historic Swedish minority that forms more than 5% of the total population (Nieminen 2000). A significant part of Finns have been born or lived abroad as a result of post-World War II labour emigration that brought tens of thousands of people to the neighbouring country Sweden and to other Nordic countries (Vaattovaara et al. 2010). Many of them returned later to Finland; return migration intensified in the 1980s. In 1990s, another group of returnees started to arrive to Finland. These were Ingrian Finns, whose ancestors had left to Northwest Russia in the seventeenth century (Kulu 2001). Mainly, Russian-speaking Ingrian Finns arrived to Finland from the former Soviet Union, and after 1991 from Russia and Estonia (Vikat and Notkola 1996). The immigration flows started to increase and diversify only in 1990s, and the trend has continued till today. By the year 2013, the size of migrant population was well over quarter a million, while close to half of them originated from countries outside Europe (Table 1).

**Table 1:** Characteristics of natives and immigrants from different countries/regions of origin, men and women, Finland 2013. *Source*: Finnish register data, authors’ calculations

Population groups by country/region	Total number	Characteristics of native and immigrant groups			Structure of immigrant population		
		Employed	Population at the prime age of union	Exogamous partnerships	Employed	Immigrants at the prime age of union	Exogamous partners of Finns
	Persons	Age 18–64, %	Age 18–40, %	Age 18–40, %	Age 18–40, %	Age 18–40, %	Age 18–40, %
All men	2,680,364	68	30	6	–	–	–
Native Finns	2,545,997	68	29	3	–	–	–
Immigrants	134,367	59	53	40	100	100	100
West	21,537	64	43	85	14	13	37
Russia	25,841	57	48	18	17	18	9
Estonia	18,710	72	49	23	16	13	7
East. Europe	12,043	65	56	28	11	10	8
Somalia	5059	31	58	10	2	4	1
Middle East	21,768	43	58	40	13	18	16
Asia	16,694	62	63	22	16	15	7
Other	12,715	61	60	62	11	11	16
All women	2,770,906	72	28	7	–	–	–
Native Finns	2,640,232	73	26	3	–	–	–
Immigrants	130,674	54	50	39	100	100	100
West	12,449	62	45	77	10	9	15
Russia	40,117	54	40	36	26	25	24
Estonia	20,308	73	42	32	18	13	10
East. Europe	10,461	58	59	28	11	10	8
Somalia	4557	20	59	4	2	4	0
Middle East	11,933	32	59	6	7	11	2
Asia	21,857	53	61	54	20	21	32
Other	8992	52	55	49	7	8	9

Exogamous partnerships are defined as partnerships between native Finns and immigrants. In this table, West signifies Western Europe, North America, Australia and New Zealand

Ethnic minorities and recent immigrants live mainly in the three main urban areas of Finland, as well as in southern and western coastal areas and areas close to Russian border in the east (Vaattovaara et al. 2010). Segregation levels within the cities have grown along with the increase in the number of new immigrants. It is important to note that the capital city Helsinki—metropolitan area that hosts more than half of the immigrant population—has so far succeeded to avoid extreme spatial concentration of migrant groups (Kauppinen and Vaalavuo 2017). Still, the tendency of migrant population being concentrated into lower-paid jobs or staying out of the labour market has increased during the economic recession in 2000s (Vaatovaara et al. 2010).

The increasing number of immigrants has had an effect on inter-ethnic partnership patterns as well (Heikkilä 2006; Leinonen 2011; Lainiala and Säävälä 2012; Heikkilä et al. 2014; Heikkilä and Rauhut 2015). In 1999, the overall proportion of inter-ethnic partnerships among Finns was 1% of all partnerships; by 2013, it had doubled for both men and women. Among Finns at the prime age of partnership formation (18–40), the proportion of inter-ethnic partnerships reached 3% (Table 1), whereas the overall proportion of immigrants in this age group had climbed to nearly 9%.

The last three columns of Table 1 show the ethnic composition of all immigrants at the prime age of partnership formation (18–40) and two sub-populations of immigrants: those who are employed and those who are living with a Finnish partner. It appears that the composition of migrants with whom Finns have formed cohabiting partnerships diverges from the overall composition of the immigrant population. Although these aggregate insights suggest that migrant groups have different integration strategies, it is not clear how the individual-level partnership choices of native Finns respond to ongoing changes in the population composition and levels of ethnic diversity experienced at places of work and residence.

## Data and Methods

Our study is based on longitudinal register data, compiled by Statistics Finland. The data set was formed through the linking of data from a population register and registers of employment and educational qualifications. The data set covers all residents who ever lived in Finland in 1999–2014. In the study, we focus only on individuals for whom we were able to construct partnership history from age 18 onwards. Thus, our research data cover individuals who were born between 1981 and 1995 (i.e. those who were 18 years old or younger in 1999 and reached age 18 before 2014). A minor group of adolescents who had started a partnership before age 18 were excluded.

Unlike many studies of ethnic intermarriage, we focus on natives instead of immigrants. We define native Finns as individuals who were born in Finland. In addition, our study population includes return migrants who were born abroad to Finnish-born parent(s) and who had moved back to their parents’ country of birth. Persons who were born in Finland to foreign-born parents (the second generation of immigrants) are also included, but given the relatively recent start of large-scale immigration to Finland, the proportion of the second-generation immigrants is relatively low in the birth cohorts covered.

The event of interest is the formation of first partnership. Based on the yearly information about the place of residence (down to the specific dwelling), co-residing conjugal partners are identifiable in the Finnish register data, even when they are unmarried and childless. In this study, we draw on the procedure employed in the register to identify partnerships. We use discrete-time data with yearly intervals; the first observation when the partners are registered in the same address is considered to indicate the beginning of their marital or cohabiting union. One of the limitations of this approach is that it underestimates the prevalence of partnerships. In particular, it misses unions where partners de facto cohabit, but are registered in different addresses. Likewise, cohabiting unions of relatively short duration (less than a year) which are formed and dissolved between two yearly observations are not considered in the analysis. For the purpose of our study, two types of partnerships are distinguished: endogamous partnerships (both partners are native Finns) and exogamous partnership (the partner is migrant). To denote the latter type of unions, we also employ the term inter-ethnic (ethnically mixed) partnership in the article. In order to account for the heterogeneity of the immigrants who have arrived in Finland, we distinguish between inter-ethnic unions with immigrant partners of Western and non-Western origin in the analysis (the details of the groupings are discussed in the following sections). After all necessary exclusions, our final research data set included 494,040 women and 518,513 men who formed 11,918 and 7501 inter-ethnic partnerships, respectively.

### Statistical Methods

We estimated proportional hazards event history models separately for native women and men. The exposure time started at age 18 and ended in the year during which the first partnership was formed. Exposure time was censored in 2014, if a person was never partnered at that time, or earlier, if a person left the country or died. In order to count the fact that union formation risks change in age, we held our baseline hazard (age) constant during the intervals of 2 years, but allowed it to vary between the intervals. For women and men alike, we estimated two sets of competing risk models, for exogamous and endogamous partnerships, respectively. For exogamous unions, additional group-specific models were estimated for partnerships with individuals of Western and non-Western origin.

### Measures of Ethnic Diversity

We calculated the share of immigrants in the area of residence. We opted for the share of migrants in age group 18–40 that focuses on the individuals who are most active in the partnership market. The neighbourhoods were defined according to postal service codes (so-called zip areas). If the zip area was not known for a person, we used the proportion of immigrants in the municipality. To avoid the overestimation of the impact of neighbourhood, we backdated the variables related to place of residence for 1 year.

The register data used in the study provided employment information for all individuals who were currently employed, including the encrypted identification numbers of the enterprise and establishment where a person worked. This allowed us to calculate the share of immigrants at the workplace. Unlike for the neighbourhood, no restriction was imposed on the age of the employees because by default the work-related information is limited to working-age population. Individuals who were currently not employed were classified either as ‘studying’ or included in the residual category (‘other/not known’). We also experimented with the backdating, but unlike for neighbourhood the results deemed it unnecessary for our work-related variables.

### Control Variables

To account for the fact that neighbourhoods and workplaces markedly vary in size, we included respective controls for both domains. The neighbourhood size is a continuous variable indicating the number of residents in the area (in logarithmic scale); a categorical specification is used for the size of workplace (establishment). In addition, the region of residence (Helsinki, Turku and Tampere represent the three largest cities of Finland, and the rest of the country is included in ‘other urban or rural areas’) was added to the controls.^1^ The purpose of this variable was to remove the variation associated with a wider context of residence from our neighbourhood variable. The daily activity space of people reaches beyond residential neighbourhoods and workplaces and often includes the whole city region. Hence, the proximity network and socialization effects can stem from a city level as well.

Individual-level controls included birth cohort, origin, experience of living abroad, mother tongue and educational attainment. The origin distinguishes between persons who were born in Finland to Finnish-born parents and persons who themselves or whose parents were born abroad. Another time-constant variable distinguishes between Finnish-speaking native majority, Swedish-speaking native minority and a small group of native Finns who have an indication of any other language as mother tongue in the register. To account for the influence of international migration, the experience of living abroad considers the episodes during which a person has resided outside Finland. Finally, we also controlled for socio-economic status by using a time-varying information about the highest level of education.

## Results

### Ethnic Diversity in Residential Neighbourhood

Table 2 shows the results from a series of proportional hazards models indicating the probability that native Finnish women and men will start first union with immigrant partner.

**Table 2:** Hazard ratios for the transition to exogamous and endogamous first partnerships by ethnic diversity in residential neighbourhood, Finland, native population, birth cohorts 1981–1995. *Source*: Finnish register data, authors’ calculations

Proportion of immigrants in residential neighbourhood, %	Women				Men			
	Exogamous partnership		Endogamous partnership		Exogamous partnership		Endogamous partnership	
	M1.1	M1.2	M1.1	M1.2	M1.1	M1.2	M1.1	M1.2
0–4 (ref.)	1	1	1	1	1	1	1	1
5–9	1.77***	1.15***	0.90***	0.93***	1.82***	1.21***	0.95***	0.85***
10–14	2.19***	1.27***	0.88***	0.94***	2.16***	1.28***	0.91***	0.79***
15 +	2.88***	1.45***	0.81***	0.90***	2.66***	1.37***	0.86***	0.72***
*Log likelihood*	− *55,348*	− *54,232*	− *548,338*	− *541,383*	− *36,972*	− *36,232*	− *477,666*	− *468,532*

***p < 0.01; **p < 0.05; *p < 0.1

Time at risk starts at age 18, censoring occurs at entry into a competing type of partnership, end of observation period, emigration or death

Model 1.1: Includes the process time and the proportion of immigrants in the residential neighbourhood

Model 1.2: Additional controls for the size of the residential neighbourhood, birth cohort, the respondents’ own and parents’ country of birth, having lived abroad, mother tongue, educational attainment and region of residence were added to Model 1.1

The initial model (M1.1) includes the ethnic diversity in the residential neighbourhood. The estimates from this model reveal a strong positive association between the share of immigrants in the neighbourhood and the propensity of native Finns to form partnerships with them. Among women, living in areas where the proportion of immigrants ranges from five to nine per cent relates to 77% increase in the likelihood of inter-ethnic union, compared to the reference category (the share of immigrants below five per cent). For men, the effect appears closely similar (+ 82%). Larger proportions of immigrants further increase the chance of forming an ethnically mixed partnership. In the areas of highest concentration of immigrants, the likelihood that native women and men would start a mixed union exceed the reference category 2.8 and 2.7 times, respectively.^2^

Further, a series of control variables were added to the model. This markedly reduced the hazard ratio for ethnic diversity in the neighbourhood (M1.2). For areas with the highest concentration of immigrants, the hazard ratio decreased from 2.88 to 1.45 among women and from 2.66 to 1.37 among men, respectively. However, despite a reduction relative to the initial model, living in an ethnically diverse neighbourhood significantly increases the likelihood of native Finns’ initiating a mixed partnership. A statistically significant increase also persists in areas with a relatively moderate (five to nine per cent) proportion of immigrants. This applies to women and men alike, with no marked gender difference in the effect.

A stepwise procedure of adding variables to the model (not shown in Table 2) revealed the controls that made a more sizeable contribution to the change in the effect of the neighbourhood variable. The inclusion of the size of the residential neighbourhood produced a moderate reduction in the hazard ratios associated with the proportion of immigrants in the area while the inclusion of the characteristics of individuals made only little difference in the relationship between ethnic diversity and the likelihood of forming a mixed partnership. By contrast, the inclusion of the region markedly reduced the hazard ratio for ethnic diversity in the neighbourhood. In our view, this indicates that local marriage markets are not confined to immediate neighbourhoods, but cover much wider geographical areas.

The results for endogamous partnerships corroborate the findings reported above. Unlike for mixed unions, the models for endogamous partnerships reveal a negative gradient for the main independent variable. Among native Finns, the ethnic diversity in residential neighbourhood is associated with significant decrease in the propensity to form endogamous unions. The comparison of results across models (M1.1 and M1.2) shows that the statistically significant negative effect does not fade away after the inclusion of controls for the size of residential area, individual characteristics and the region in the models. The opposite gradient of our main independent variable for endogamous and exogamous partnerships suggests that the increase in ethnic diversity in residential neighbourhoods has a potential of advancing the ethnic intermarriage and replacing endogamous unions in part with ethnically mixed partnerships.

### Ethnic Diversity at Workplace

In order to get an account of the effects on partnership formation of ethnic diversity at work, another set of event history models was estimated (Table 3). In these models, the main independent variable is the share of co-workers of immigrant background at the person’s place of work.^3^

**Table 3:** Hazard ratios for the transition to exogamous and endogamous first partnerships by ethnic diversity at workplace, Finland, native population, birth cohorts 1981–1995. *Source*: Finnish register data, authors’ calculations

Proportion of immigrants at workplace, %	Women				Men			
	Exogamous partnership		Endogamous partnership		Exogamous partnership		Endogamous partnership	
	M2.1	M2.2	M2.1	M2.2	M2.1	M2.2	M2.1	M2.2
0–4 (ref.)	1	1	1	1	1	1	1	1
5–9	1.51***	1.24***	0.91***	0.97***	1.46***	1.16***	0.95***	0.95***
10–14	1.92***	1.46***	0.88***	0.96***	1.85***	1.40***	0.92***	0.92***
15 +	2.53***	1.77***	0.82***	0.91***	2.27***	1.57***	0.87***	0.89***
*Log likelihood*	− *55,720*	− *54,186*	− *543,943*	− *539,720*	− *37,133*	− *36,146*	− *467,419*	− *464,392*

***p < 0.01; **p < 0.05; *p < 0.1

Time at risk starts at age 18, censoring occurs at entry into a competing type of partnership, end of observation period, emigration or death

Model 2.1: includes process time and the proportion of immigrants in the workplace

Model 2.2: Additional controls for the size of the workplace, birth cohort, the respondents’ own and parents’ country of birth, having lived abroad, mother tongue, educational attainment and region of residence were added to Model 2.1

The initial model (M2.1) includes the ethnic diversity at workplace. The estimates from the initial model indicate a significant positive relationship between the share of immigrant co-workers and the propensity of native Finns to enter ethnically mixed unions. For women, having a workplace in which the proportion of immigrants ranges from five to nine per cent of employees is associated with 51% increase in the hazard ratio relative to the reference category (workplaces with less than five per cent of immigrants). Among men, the relationship is closely similar (46% increase in the hazard ratio). Larger proportions of co-workers with immigrant background further add to the likelihood of forming an ethnically mixed partnership in more or less a linear fashion. In case the share exceeds 15%, native women and men in Finland exhibit a 2.5- and 2.3-fold increase in the chances of partnering with a person of non-native background, respectively.

The addition of the control variables to the model markedly reduced the hazard ratio for ethnic diversity at workplace (Model M2.1). For workplaces with the highest proportion of immigrants, the hazard ratio decreased from 2.53 to 1.77 among women and from 2.27 to 1.57 among men, respectively. However, notwithstanding the change related to the inclusion of the control variables, Model M2.1 clearly indicates that ethnically diverse workplaces are associated with an elevated likelihood of native Finns’ initiating an exogamous union. A significant increase in the likelihood of inter-ethnic unions also persists for workplaces with a moderate (five to nine per cent) proportion of immigrants.

The stepwise addition of the control variables to the model (not shown in Table 3) identified those that made a more important contribution to the reduction in the effect of the workplace. In accord with the findings for residential neighbourhood, the inclusion of the region significantly reduced the hazard ratios for ethnic diversity in the workplace. This suggests that the hazard ratios for ethnic diversity in the workplace may partially reflect exposure to immigrants beyond the workplace, which is more common in cities, especially the capital region.

The hazard ratios for ethnic diversity at workplace appear somewhat higher for women. Although the contrast is not large, it cuts across all models and levels of the independent variable. Perhaps, this can be regarded as yet another sign of the advanced equity of men and women achieved in the Nordic societies, with work being an equally important part of people’s lives, irrespective of gender.

For endogamous unions, the association with ethnic diversity at work runs in the opposite direction: the increase in proportion of immigrants at workplace leads to lower propensity to start endogamous partnerships. According to the final model (M2.2), native men exhibit up to 11% decrease in the rate of entry into endogamous partnerships, associated with the increase in immigrants at workplace. Among women, the reduction in the hazard ratio is only slightly smaller (− 9%). Across models, the hazard ratios for endogamous unions are more stable than those observed for exogamous partnerships. This lends support to the notion that the control variables employed in the analysis are more extensively modulating the formation of the latter kind of partnerships. The opposing gradients found for exogamous and endogamous partnerships suggest that the increase in ethnic diversity at workplaces tends to promote intermarriage between natives and immigrants.

### Joint Effects of the Ethnic Diversity in Neighbourhoods and Workplaces

In the two previous sections, the role of ethnic diversity in residential neighbourhoods and workplaces was investigated separately. However, the contacts and integration between the natives and immigrants in different life domains do not develop in isolation. To provide insight into how the inter-ethnic encounters occurring in neighbourhoods and workplaces come together, we estimated additional models with ethnic diversity in both domains considered (Table 4).

**Table 4:** Hazard ratios for the transition to exogamous and endogamous first partnerships by ethnic diversity in neighbourhood and workplace, Finland, native population, birth cohorts 1981–1995. *Source*: Finnish register data, authors’ calculations

Proportion of immigrants, %	Women		Men	
	Exogamous partnership M3	Endogamous partnership M3	Exogamous partnership M3	Endogamous partnership M3
In residential neighbourhood				
0–4 (ref.)	1	1	1	1
5–9	1.13***	0.93***	1.21***	0.86***
10–14	1.24***	0.93***	1.27***	0.80***
15 +	1.40***	0.89***	1.33***	0.73***
At workplace				
0–4 (ref.)	1	1	1	1
5–9	1.23***	0.97***	1.15***	0.95***
10–14	1.43***	0.96***	1.37***	0.93***
15 +	1.73***	0.92***	1.53***	0.90***
*Log likelihood*	− *54,061*	− *537,270*	− *36,047*	− *457,680*

***p < 0.01; **p < 0.05; *p < 0.1

Time at risk starts at age 18, censoring occurs at entry into a competing type of partnership, end of observation period, emigration or death

Model 3: An additional control for the proportion of immigrants in the residential neighbourhood was added to Model 2.2 (Table 3)

The estimates for the control variables are presented in Table 7

The estimates obtained from the joint model (M3) show that the effects of ethnic diversity in residential neighbourhood and workplace are only slightly altered compared to the separate models (M1.2 and M2.2 in Tables 2 and 3). For exogamous partnerships, the largest reduction in the hazard ratio (from 45 to 40%) can be observed among women residing in the neighbourhoods with high share of immigrants. Otherwise, the typical change in the hazard ratios is limited to 2–3 percentage points; for endogamous unions, the change is even smaller. A tentative conclusion that can be drawn is that the influence of ethnic diversity in residential neighbourhoods and workplaces is to a large extent independent from each other.

The results obtained from the joint models also suggest that the increase in ethnic diversity at workplaces exerts somewhat stronger influence on the propensity to start exogamous partnerships than diversity at residential neighbourhoods. For both women and men, the hazard ratios for the share of immigrant co-workers in most cases exceed those for the proportion of immigrants in the neighbourhood.^4^

To further elaborate the interplay of ethnic diversity in different domains, we employed an interaction between our two main independent variables (Table 5). In the interaction model, we distinguished between residential neighbourhoods and workplaces with higher (10% or above) and lower (below 10%) share of immigrants. To facilitate the interpretation of the results, we have derived two additional sub-tables (the middle and lower panels) from the primary interaction (the upper panel).

**Table 5:** Interaction of ethnic diversity in neighbourhood and workplace for the transition to exogamous first partnerships, Finland, native population, birth cohorts 1981–1995

Proportion of immigrants in residential neighbourhood, %	Women		Men	
	Proportion of immigrants at workplace, %		Proportion of immigrants at workplace, %	
	M4		M4	
	0–9	10 +	0–9	10 +
Upper panel: primary interaction				
0–9	1	1.60***	1	1.56***
10 +	1.20***	1.71***	1.20***	1.50***
Middle panel: the impact of ethnic diversity in the neighbourhood				
0–9	1	1	1	1
10 +	1.20***	1.07***	1.20***	0.97*
Lower panel: the impact of ethnic diversity at the workplace				
0–9	1	1.60***	1	1.56***
10 +	1	1.43***	1	1.25***
*Log likelihood*		− *54,121*		− *36,072*

***p < 0.01; **p < 0.05; *p < 0.1

Time at risk starts at age 18, censoring occurs at entry into a competing type of partnership, end of observation period, emigration or death

Model 4: The controls included in the model are as indicated for M3 in Table 4

The middle panel of the table shows how the chance of starting a mixed partnership is modulated by the ethnic diversity in the neighbourhood for native women and men who are employed in establishments with lower and higher share of immigrants, respectively. The high ethnic diversity in neighbourhood makes a larger contribution among people who encounter less diversity at work; both women and men belonging to the latter group exhibit a 20% increase in the likelihood of exogamous union. By contrast, among those who are more exposed to inter-ethnic contacts at workplace, the role of ethnic diversity in the neighbourhood appears more limited. For women, it adds seven per cent to the likelihood of partnering with immigrants. For men more exposed to inter-ethnic contacts at workplace, living in ethnically diverse neighbourhood fails to make any additional contribution to mixed partnerships.

The lowermost panel of Table 5 illuminates the ways how the forming of exogamous partnerships is shaped by the ethnic diversity at workplace among natives who are living in the neighbourhoods with varying proportion of immigrants. In our view, two main conclusions can be drawn from the panel. First, the effect of ethnic diversity at workplace appears more pronounced among persons who live in neighbourhoods with lower share of immigrants. This corroborates an observation made in the previous paragraph for the area of residence. Viewed together, these findings tell us that the effect of inter-ethnic encounters on partnership formation may not be (fully) multiplicative across life domains. The increased exposure to ethnic diversity in one domain seems to reduce the relative contribution of additional inter-ethnic contacts that occur in other domains. Second, the results lend further support to the notion derived from the main effects models that the share of immigrants at workplaces has a more significant bearing on the formation of native-immigrant partnerships than the ethnic diversity in residential neighbourhoods. We think that the latter result plausibly arises from the greater intensity of interpersonal encounters at workplaces relative to those that occur in residential neighbourhoods.

Finally, the results from interaction models suggest that the effect of ethnic diversity is somewhat more pronounced among women. This can be observed in the lowermost panel of Table 5, which presents the effect of ethnic diversity at workplace. Notably, this finding runs counter the traditionalist expectation according to which work should occupy a more central role in the lives of men.

### Heterogeneity Associated with the Partner’s Origins

The purpose of extending the analysis in this direction was to ascertain whether the effects of ethnic diversity in residential neighbourhoods and workplaces reported earlier in the article are universal or driven by immigrants who are culturally close to the host country. In order to answer this question, additional competing risk models were estimated for exogamous unions between native Finns and immigrants from Western and non-Western countries.^5^ In these models, we reconfigured the main independent variables so that they reflect the group-specific proportions of immigrants of Western and non-Western origin in residential neighbourhoods and workplaces rather than the overall proportion of immigrants.

The upper part of Table 6 shows strong associations between the proportions of immigrants of different origins in the residential neighbourhood and the propensity of native Finns to partner with them. However, the associations seem to be group-specific. A higher proportion of immigrants of Western origin in the neighbourhood is related to a greater likelihood of forming partnerships with them. For areas with the highest proportion of Western immigrants, the group-specific hazard ratios are 1.85 for Finnish women and 2.32 for men. An elevated proportion of immigrants of non-Western origin in the neighbourhood is also associated with a higher propensity to partner with them, but the effect is somewhat less pronounced than that observed for immigrants from Western countries. In areas with the highest concentration of non-Western immigrants, the hazard ratios are 1.59 for native women and 1.61 for men.

**Table 6:** Hazard ratios for the transition to exogamous first partnerships with Western and non-Western immigrants, by ethnic diversity in the neighbourhood and at workplace, Finland, native population, birth cohorts 1981–1995. *Source*: Finnish register data, authors’ calculations

Percentage of immigrants by origin, %	Women		Men	
	Exogamous partnerships		Exogamous partnerships	
	Western partner	Non-Western partner	Western partner	Non-Western partner
	M5	M5	M5	M5
*In residential neighbourhood*				
Western immigrants				
0–4 (ref.)	1	1	1	1
5–9	1.15***	0.93*	1.17***	0.87**
10–14	1.25***	1.01	1.74***	0.95
15 +	1.85***	1.05	2.32***	0.73*
Non-Western immigrants				
0–4 (ref.)	1	1	1	1
5–9	0.94	1.33***	0.83***	1.14**
10–14	0.86**	1.41***	0.72***	1.33***
15 +	0.82	1.59***	0.52***	1.61***
*At workplace*				
Western immigrants				
0–4 (ref.)	1	1	1	1
5–9	1.20***	1.03	1.19***	0.96
10–14	1.54***	1.05	1.32***	1.09
15 +	1.89***	1.12	1.74***	1.01
Non-Western immigrants				
0–4 (ref.)	1	1	1	1
5–9	1.03	1.47***	0.94	1.53***
10–14	0.98	1.68***	0.97	1.89***
15 +	1.19**	2.42***	1.03	2.02***

***p < 0.01; **p < 0.05; *p < 0.1

Time at risk begins at age 18, censoring occurs at entry into another type of partnership, the end of the observation period, emigration, or death

Western origin refers to the countries of Europe, the USA, Canada, Australia and New Zealand. Non-Western origin refers to all other countries

Model 5: The controls included in the models are as indicated for Model M3 in Table 4

The estimates for the control variables are presented in Table 7

The lower part of Table 6 reveals significant associations with regard to the proportion of immigrants of Western and non-Western origin in the workplace. For both groups, a higher concentration of immigrants tends to increase the likelihood of partnering with native Finns. However, unlike residential neighbourhood, the group-specific effect of ethnic diversity in the workplace appears somewhat stronger for immigrants from non-Western countries. When the proportion of non-Western immigrants exceeds 15% in the workplace, the chance of their partnering with native Finnish women and men increases by 2.4 and 2.0, respectively.

The hazard ratios for immigrant groups that are beyond the focus of the particular group-specific model show a negative or neutral gradient with regard to our main independent variables.^6^ A higher proportion of non-Western immigrants in the neighbourhood is associated with a decrease in the propensity of native Finns, both women and men, to partner with those of Western origin. Likewise, a high proportion of Western immigrants tends to reduce the likelihood of Finnish men’s partnering with women of non-Western origin. The pattern among Finnish women is less clear. These results support the notion that different immigrant groups may to some extent be competitors in the local marriage market. However, this pattern does not extend to the workplace, where an elevated proportion of potential partners from another immigrant group does not in most cases have a statistically significant effect.

In summary, our results are at odds with the expectation that the effects of ethnic diversity in residential neighbourhoods and workplaces reported in the previous sections are primarily driven by immigrants who are closer to Finns. The findings suggest that encounters in neighbourhoods and workplaces may increase the likelihood of exogamous partnerships both for groups who are more similar to the host society and for those who are more distant.

### Effects Associated with Control Variables

The estimates for control variables presented in Table 7 are obtained from models that jointly consider the effect of ethnic diversity in residential neighbourhoods and workplaces for the formation of exogamous unions (M3 in Table 4, and M5 in Table 6). In addition to the overall pattern, the differences between unions with Western and non-Western migrants are also addressed.

**Table 7:** Hazard ratios of control variables for the transition to exogamous first partnerships with Western and non-Western immigrants, by control variables, Finland, native population, birth cohorts 1981–1995. *Source*: Finnish register data, authors’ calculations

Control variable	Women			Men		
	Exogamous partnerships			Exogamous partnerships		
	All partners M3	Western partner M5	Non-Western partner M5	All partners M3	Western partner M5	Non-Western partner M5
Size of neighbourhood (ln)	1.11***	1.09***	1.12**	1.12***	1.13***	1.14***
Size of workplace						
1–4	1	1	1	1	1	1
5–29	0.90***	0.89***	0.95	0.97	0.99	0.97
30–99	0.86***	0.88***	0.92	0.94	0.95	0.99
100 +	0.84***	0.86***	0.91	0.95	0.92	1.15
Region of residence						
Helsinki	1	1	1	1	1	1
Turku	0.74***	0.70***	0.71***	0.80***	0.80***	0.63***
Tampere	0.81***	0.80***	0.73***	0.94	0.94***	0.81***
Other areas	0.51***	0.52***	0.40***	0.55***	0.55***	0.41***
Birth cohort						
1981–1985	1	1	1	1	1	1
1986–1995	1.18***	1.19***	1.23***	1.14***	1.16***	1.21***
Origin						
Born in Finland, parent(s) born in Finland	1	1	1	1	1	1
Born abroad, parent(s) born in Finland	1.23***	1.19***	1.30***	1.39***	1.32***	1.54***
Born in Finland, parent(s) born abroad	2.60***	1.68***	3.67***	2.40***	1.23	5.29***
Experience of living abroad						
No	1	1	1	1	1	1
Yes	2.42***	3.10***	1.41***	2.55***	2.79***	2.91***
Mother tongue						
Finnish	1	1	1	1	1	1
Swedish	1.24***	1.37***	1.03	1.28***	1.39***	1.00
Other	1.73***	1.77***	1.62***	2.22***	2.28***	2.17***
Education						
ISCED1–2	1.10***	1.01	1.22***	1.06*	1.07*	1.03
ISCED3–4	1	1	1	1	1	1
ISCED5 +	1.15***	1.22***	1.05	1.48***	1.51***	1.45***
*Subjects*	*494,040*	*494,040*	*494,040*	*518,513*	*518,513*	*518,513*
*Events*	*11,918*	*7079*	*4839*	*7501*	*5063*	*2438*
*Person years*	*2,232,064*	*2,232,064*	*2,232,064*	*2,904,677*	*2,904,677*	*2,904,677*

***p < 0.01; **p < 0.05; *p < 0.1

Time at risk starts at age 18, censoring occurs at entry into a competing type of partnership, end of observation period, emigration or death

The estimates for the main independent variables are presented in Table 4 (Model M3) and Table 6 (Model M5)

The size of residential neighbourhood features a similar gradient across models. The increase in the number of residents in the area evidently reflects an expanding pool of potential partners available for single women and men. The effects are largely similar for partnerships formed with immigrants of Western and non-Western origin. The size of workplace shows less systematic pattern. For women, a larger number of co-workers moderately reduces the likelihood of partnering with immigrants of Western origin. However, for partnerships with immigrants of non-Western origin, the size of the workplace exerts no significant influence. For men, the effects are insignificant irrespective of the partner’s origins.

Region of residence has a strong bearing on the formation of exogamous unions. Not surprisingly, the likelihood of partnering with immigrants is highest in Helsinki, followed by Turku and Tampere, the two largest urban centres besides the capital region. The pattern is similar for unions with Western and non-Western partners. Since immigration on a larger scale is a relatively recent phenomenon in Finland, younger women and men have had more opportunities to meet a non-native partner. Despite that, our analysis covers a relatively narrow range of cohorts, women and men born in late 1980s and early 1990s show a significantly higher likelihood of forming inter-ethnic partnerships than their counterparts in generations born in early 1980s. The increase can be observed for partnerships between native Finns and immigrants of Western as well as non-Western origin.

Being born in Finland to foreign-born parents markedly increases the chance of forming an exogamous union. For these women and men, the hazard ratios for mixed partnerships amount to 2.6 and 2.4 times over the reference category, respectively. The effect appears more pronounced for unions with non-Western partners. This probably reflects a lesser likelihood of partnering with native Finns among the descendants of non-Western immigrants in Finland, compared with the offspring of Western immigrants. It is also interesting to note that the second-generation immigrants feature a much stronger inclination towards exogamous partnerships than return migrants of Finnish origin who were born abroad to Finnish parent(s). In our view, this finding once again underlines the role of individual’s family origin for the partner choice. The effect appears stronger for unions with non-Western partners.

Further, the experience of living abroad for some period makes a remarkably strong contribution to the likelihood of exogamous unions. For men, this experience implies a 2.6-fold increase in the likelihood of mixed partnering, and for women the hazard ratio appears slightly smaller. Unlike family background, living abroad exerts a stronger influence on the propensity to form exogamous unions with partners of Western origin. We assume that this is related to the fact that native Finns migrate predominantly to Western countries. However, we are not able to establish the exact causality here. For instance, migration to a foreign country may be itself driven by partnership formation. In addition, experience of living abroad may also involve selection as international migrants do not constitute a random subgroup of the sending population (Ng and Nault 1997; Frank and Heuveline 2005). If they are selected for greater openness to inter-ethnic contacts, this can reinforce the observed relationship beyond causality. Mother tongue other than Finnish also relates to significantly elevated chance of starting an exogamous union and reduced likelihood of having a native partner. The contrast with the reference group (persons speaking Finnish as mother tongue) is more pronounced for those non-Finnish speakers who do not belong to the Swedish-speaking population. This result is not surprising because the majority of Swedish speakers come from an historical minority which has lived in Finland for many centuries. The increased likelihood of Swedish speakers’ forming partnerships with those of Western origin plausibly reflects their higher propensity to partner with Swedes.

Finally, the relationship between the ethnically mixed unions and educational attainment follows a U-shaped pattern. Judging from hazard ratios, the inclination towards partnerships with immigrants is lowest among women and men with medium level of education. By contrast, the association of schooling with endogamous unions follows a linear pattern. The results from the group-specific models, estimated for inter-ethnic unions with partners of Western and non-Western origin, reveal that a U shape is produced by amalgamating two different patterns. Finnish women with low educational attainment are more likely than their better educated peers to partner with immigrants from non-Western countries. By contrast, highly educated Finnish women exhibit a significantly greater propensity to form unions with men from Western countries. However, this pattern is not characteristic of Finnish men. For the latter, an elevated risk of inter-ethnic union is associated with high educational attainment, regardless of the partner’s origins.

## Summary and Discussion of the Findings

Taking advantage of the high-quality longitudinal data from registers, the main focus of this study lies with the role of two domains—residential neighbourhood and workplace—in the formation of first unions among native Finns born after 1980. To the best of our knowledge, this is one of the few studies of ethnic intermarriage that considers both the workplace and the neighbourhood context. To analyse the impact of interpersonal encounters in these domains on the partner choice, we estimated a series of proportional hazards models that distinguish between inter-ethnic unions with foreign-born partners and endogamous unions with native Finns. In the context of Nordic countries, the native perspective on ethnic intermarriage has not been frequently applied, despite the fact that for inter-ethnic unions to occur, the structure of native marriage market is as important factor as are the integration strategies of migrant groups.

The results generally support our *first hypothesis* that a higher proportion of immigrants in the residential neighbourhood and workplace both makes a marked contribution to choosing a foreign-born partner. However, the analysis did not corroborate our *second hypothesis,* which anticipated that neighbourhoods and workplaces with the highest proportion of immigrants would reduce the willingness of natives to engage in inter-ethnic unions. In other words, our findings on native Finns do not conform to the group threat theory (Blalock 1967; Quillian 1995) according to which the increase in the share of members of the other group might stimulate negative feelings and reduce the likelihood of inter-group partnerships. However, the lack of empirical support does not necessarily prove the hypothesis fundamentally wrong. It can be speculated that perhaps the concentration of immigrants in Finland has not yet reached the levels beyond which the expected pattern may emerge. The observed inverse association between endogamous unions and the share of immigrants in the neighbourhood and workplace suggests that the presence of immigrants tends to substitute part of the endogamous unions with ethnically mixed partnerships among the host country natives. In the policy perspective, the findings lead to conclusion that the decrease in residential and workplace segregation can pave the way towards more fundamental forms of immigrant integration, such as intermarriage (Kalmijn and van Tubergen 2010; Logan and Shin 2012).

Judging from models estimated separately for either domain, living in areas with high concentration of immigrants was found to involve up to two-fifth increase in the chance of exogamous unions; the presence of immigrants at workplace makes an even larger contribution. The latter result is in line with our *third hypothesis* which drew its argument from the greater intensity of interpersonal contacts occurring at workplaces, as compared to those in the neighbourhoods. Further evidence that the share of immigrants in workplace has more important bearing on the partner choice came from the interaction model. This finding adds to the evidence obtained in previous studies that the chances for natives to meet immigrants are higher at places of work than in neighbourhoods (Ellis et al. 2004; Strömgren et al. 2014). As regards, mechanisms underpinning the observed result, one could think on selectivity as immigrants who are employed tend to be better integrated to host society than their non-employed counterparts. In the policy context, these findings draw attention to indirect benefits of the labour market integration of immigrants. The interaction models also illuminated how the effects of ethnic diversity come together across life domains. According to our study, the effects of neighbourhood and workplace contexts are not fully multiplicative: the higher exposure to inter-ethnic contacts in one domain tends to be associated with reduced effect of the additional exposure in another domain.

The consideration of both women and men provided this study with a systematic gender perspective which is not always present in the analyses of inter-ethnic partnership formation. We expected that residential context is more important for native women (*fourth hypothesis)*, while workplace context appears more important for men (*fifth hypothesis).* At odds with our expectation, neither of these hypotheses was supported by the results. The ethnic diversity at workplace was found to have a more pronounced effect on partnering choices among women. Although the difference is not large, it appears to be systematic and cut across all models and levels of the independent variable. We are inclined to relate this result to contextual features: the manifestation of advanced gender equity characteristic of the Nordic-type societies.

A counter-hypothesis anticipated disappearance of the effect of ethnic concentration after controlling for contextual and individual characteristics (*sixth hypothesis*). This was not the case. Although reduced in magnitude, statistically significant positive effects persisted after the inclusion of individual and contextual controls in the models. Still, a variety of associations between control variables and choice of partner deserve attention. Evidently owing to larger pool of potential partners, the size of neighbourhood was found to make a systematically positive contribution to union formation, with natives and immigrants alike. Across regions, partnering with immigrants appears expectedly most common in Helsinki, followed by other large urban centres, in which inter-ethnic encounters are more frequent than in the rest of the country.

We also investigated whether the associations between the residential and workplace context and inter-ethnic partnering vary according to the type of exogamous partnership. The expectation was that these associations would be more pronounced in the case of partners from regions that are culturally closer to the host society (*seventh hypothesis*). This expectation was not supported by the results. The effects of the proportion of immigrants of Western and non-Western origin in the residential neighbourhood were not markedly different.^7^ However, the control variables in the models revealed some interesting differences in the group-specific patterns of intermarriage. In accord with the predictions of the status exchange theory, Finnish women with low educational attainment featured a greater propensity to partner with immigrants from non-Western countries. Women with higher education, on the other hand, were more likely to partner with immigrants from Western countries. However, this pattern was not characteristic of Finnish men, among whom an elevated risk of exogamous union was associated with higher education, regardless of the origins of the partner. This finding underscores the relevance of heterogeneity among immigrant groups and suggests that the dynamics of status exchange might differ by gender.

The study also yielded results that are noteworthy from the methodological point of view. The experience gained underscores the importance of backdating the independent variables that relate to residential context when using discrete-time longitudinal data for the analysis of union formation. According to our results, the failure to do so tends to result in an overestimation of the effect associated with the share of immigrants in residential neighbourhoods. The overestimation arises from the fact that migration is often driven by family formation, a situation termed ‘interrelation of events’ in the literature (Andersson 2004; Kulu and González-Ferrer 2014). In order to account for the fact that the partnership market is not limited to Finland, we reran our models excluding imported partners. The latter were defined as immigrants who, after arriving in the host country, began living with a partner without having lived alone. The additional models (available from the authors) confirmed the robustness of our main findings with regard to the role of inter-ethnic encounters in the workplace and residential neighbourhood.

Finally, this study is not without limitations. First, notwithstanding the highly accurate longitudinal data on neighbourhood and workplace context provided the registers, the evidence is wholly lacking on other important elements, especially preferences, which are known to shape individuals’ decisions with regard to partner choice (Kalmijn 1998). Second, although neighbourhood and workplace belong to most profoundly researched life domains in the intermarriage literature, there are still other domains (schools, recreational places, transport) which are not considered in the analysis (Van Ham and Tammaru 2016). Third, although we find that the immediate context is important in facilitating inter-ethnic unions between natives and immigrants, we do not know where partners actually meet. As a result, we were not able to disentangle the role of proximity, network and socialization mechanisms in our analysis. Fourth, our study applies a one-sided approach and focuses only on the natives. It follows that the results reported above cannot be transferred to immigrants who have settled in Finland, at least not without reservation. However, we believe that these shortcomings do not invalidate main the conclusions obtained in this study. We expect that part of the existing limitations can be addressed in our future research.

## Footnotes

1 During revision of the article, we estimated models with a more detailed breakdown of regional units. However, as this finer dissagregation did not alter the observed pattern for our main independent variables, it is not used in the final version.

2 The area of residence is backdated for one year in order to avoid reverse causality between migration and union formation. Additional models (available on request) show that the failure to backdate the independent variable may result in a significant (roughly twofold) overestimation of the effect of the proportion of immigrants in area of residence. The control variables for the size of neighbourhood and region are also backdated for 1 year.

3 Unlike for residential neighbourhood, the estimates for workplace did not markedly change when the variable was backdated. Likewise, there was no substantial difference in the results depending on whether the workplace based on the concept of enterprise (legal business unit) or establishment (local activity unit). At the preliminary stage, we also estimated the interaction between the size of the workplace and the proportion of immigrant workers. Significant but largely similar effects of the presence of immigrants were observed for both smaller and larger workplaces.

4 The only exception from the prevailing pattern relates to men who are living in areas and working in establishments with a moderate proportion (5–9%) of persons with immigrant background.

5 Western origin refers to all countries of Europe (including Russia as a neighbouring country to Finland), the United States, Canada, Australia, and New Zealand. In broad terms, these countries are relatively closer to Finland in terms of culture, geographical location and/or standard of living. Partners from other countries were included in the alternative group (non-Western origin). To a large extent this group is comprised of countries geographically and culturally more distant from Finland and with lower standards of living (out of all non-Western partners included in the analysis 91% of women and 97% of men came from countries where the GDP in the year 2000 was less than 50% of that of Finland).

6 The only exception is a positive effect associated with a high concentration of non-Western immigrants in the workplace in the model for partnering between Finnish women and immigrants from Western countries.

7 Our results do not preclude specificities associated with immigrants from certain countries. However, the relatively small number of unions between native Finns and immigrants prevented us from applying the analysis to individual countries.
